# Involvement of the miR-363-5p/P2RX4 Axis in Regulating Schwann Cell Phenotype after Nerve Injury

**DOI:** 10.3390/ijms222111601

**Published:** 2021-10-27

**Authors:** Eun-Jung Sohn, Yun-Kyeong Nam, Hwan-Tae Park

**Affiliations:** 1 Department of Molecular Neuroscience, College of Medicine, Dong-A University, Busan 602-714, Korea; handful-lo@daum.net (Y.-K.N.); phwantae@dau.ac.kr (H.-T.P.); 2 School of Medicine, Pusan National University, Yangsan 50612, Korea; 3 Department of Korean Medical Science, School of Korean Medicine, Pusan National University, Yangsan 50612, Korea

**Keywords:** microRNA, Wallerian degeneration, Schwann cells, miRNA 363-5p

## Abstract

Although microRNAs (miRNAs or miRs) have been studied in the peripheral nervous system, their function in Schwann cells remains elusive. In this study, we performed a microRNA array analysis of cyclic adenosine monophosphate (cAMP)-induced differentiated primary Schwann cells. *KEGG* pathway enrichment analysis of the target genes showed that upregulated miRNAs (mR212-5p, miR335, miR20b-5p, miR146b-3p, and miR363-5p) were related to the calcium signaling pathway, regulation of actin cytoskeleton, retrograde endocannabinoid signaling, and central carbon metabolism in cancer. Several key factors, such as purinergic receptors (P2X), guanine nucleotide-binding protein G(olf) subunit alpha (GNAL), P2RX5, P2RX3, platelet-derived growth factor receptor alpha (PDGFRA), and inositol 1,4,5-trisphosphate receptor type 2 (ITPR2; calcium signaling pathway) are potential targets of miRNAs regulating cAMP. Our analysis revealed that miRNAs were differentially expressed in cAMP-treated Schwann cells; miRNA363-5p was upregulated and directly targeted the P2X purinoceptor 4 (P2RX4)-UTR, reducing the luciferase activity of P2RX4. The expression of miRNA363-5p was inhibited and the expression of P2RX4 was upregulated in sciatic nerve injury. In contrast, miRNA363-5p expression was upregulated and P2RX4 expression was downregulated during postnatal development. Of note, a P2RX4 antagonist counteracted myelin degradation after nerve injury and increased pERK and c-Jun expression. Interestingly, a P2RX4 antagonist increased the levels of miRNA363-5p. This study suggests that a double-negative feedback loop between miRNA363-5p and P2RX4 contributes to the dedifferentiation and migration of Schwann cells after nerve injury.

## 1. Introduction

Schwann cells, major glial cells of the peripheral nervous system (PNS), undergo biochemical and morphological alterations after nerve injury, following which the distal stump of the peripheral nerve sheds myelin-derived fragments and undergoes axonal degeneration, known as Wallerian degeneration. During this stage, macrophages migrate to the injured site, and Schwann cells terminate transcription factor activity in the myelination program to express myelin protein zero (MPZ), myelin basic protein (MBP), and *Krox20/Egr-2* [1].

Purinergic receptors (P2X) are ligand-gated ion channels that respond to ATP binding, enabling ions (K^+^, Na^+^, and Ca^2+^) to cross the cell membrane [2]. Seven purinergic P2X receptor subunits (P2X1–7) have been identified in several tissue and cell types, in both humans and mice [3]. P2X purinoceptor 4 (P2RX4) plays important roles in several biological processes, including inflammatory pain [4], neuropathic pain following nerve injury [5,6], T-cell migration [7], and cardiac function [8]. P2RX4 also contributes to the fate and survival of activated microglia [9] and to memory impairment in rats with type 2 diabetes mellitus [10].

MicroRNAs (miRNAs, miRs) are small, noncoding RNA molecules that participate in post-transcriptional gene silencing by forming RNA-induced silencing complexes (RISCs), which are involved in multiple biological processes associated with cancer, aging, and differentiation [11,12]. In addition, miRNAs are involved in neuropathic pain after nerve injury [13], peripheral nerve regeneration [14], and motor neuron injury [15]; miRNAs also may be responsible for transcriptional regulation during the dedifferentiation of Schwann cells after nerve injury [16], and play a role in Schwann cell development and myelination [17,18].

In the present study, Kyoto Encyclopedia of Genes and Genomes (KEGG) pathways with upregulated miRNAs (miR 212-5p, miR 335, miR 20b-5p, miR 146b-3p, and miR 363-5p) involved in the calcium signaling pathway were examined. Purinergic P2X receptors such as P2RX5, P2RX4, and P2RX3 (in the calcium signaling pathway) were potential targets of miRNAs regulating cAMP. Therefore, we investigated the role of miRNA 363-5p in the demyelination and migration of Schwann cells via the regulation of P2RX4 expression. Blocking P2RX4 delayed demyelination by regulating extracellular signal-regulated protein kinase (ERK) signaling in vitro and in vivo. Furthermore, blocking P2RX4 elevated the expression of miRNA 363-5p. Thus, our data suggest that a double-negative feedback loop between miRNA 363-5p and P2RX4 regulates the demyelination and migration of Schwann cells.

## 2. Results

### 2.1. Functional Analysis of miRNAs in cAMP-Induced Schwann Cells

**Figure 1:**
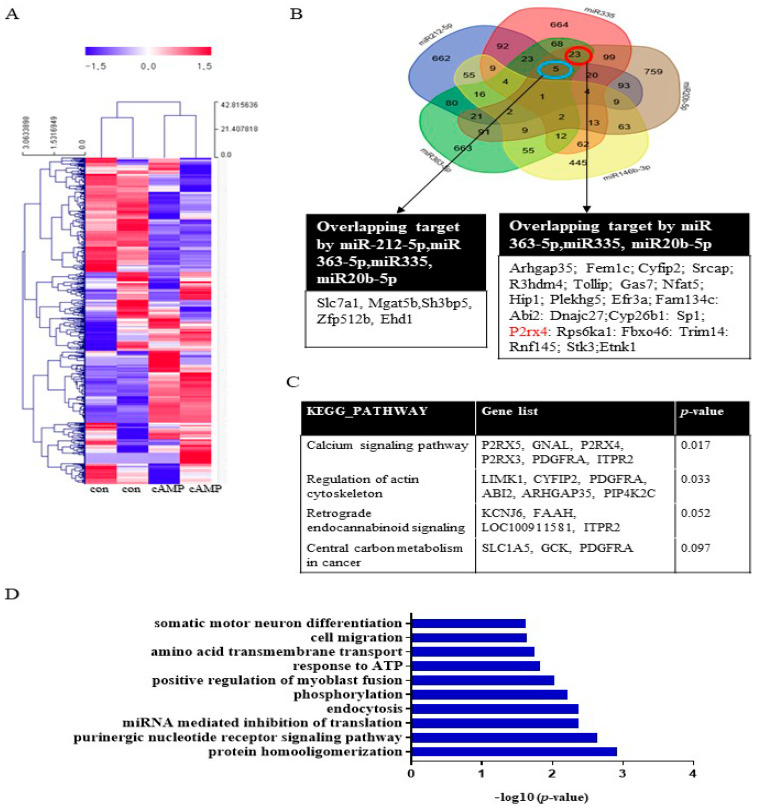
The biological functions of miRNAs in cAMP-induced Schwann cells. (**A**) miRNA expression in Schwann cells after cAMP treatment based on microarray analysis. Red indicates upregulated miRNAs; blue indicates downregulated miRNAs. *n* = 2 per sample. The heat map visualizes the hierarchical clustering of differentially expressed miRNAs (log2FC) from cAMP-treated primary Schwann cells compared with controls, according to the indicated color scale. (**B**) A Venn diagram was used to identify overlapping targets of five upregulated miRNAs (miR212-5p, miR335, miR20b-5p, miR146b-3p, and miR363-5p). *KEGG* pathways (**C**) and GO terms (**D**) for the target genes are shown.

To investigate miRNA expression during Schwann cell differentiation, Schwann cells were treated with dibutyryl cyclin adenosine triphosphate (cAMP), an inducer of differentiation, prior to miRNA analysis. The miRNA array analysis revealed that 47 miRNAs, including miRNA20b-5p, miRNA146b-3p, miRNA 363-5p, and miRNA335, were significantly upregulated (>2-fold) in cAMP-treated Schwann cells compared with the untreated control (Figure 1A, Supplementary Table S1). Conversely, 65 miRNAs were significantly downregulated (<0.5-fold), including miRNA139-3p, miRNA 242-5p, and miR138-5p. A Venn diagram was used to identify targets that overlapped among the highly expressed miRNAs (miR212-5p, miR335, miR20b-5p, miR146b-3p, and miR363-5p) (Figure 1B). The *KEGG* pathways associated with these target genes included the calcium signaling pathway, regulation of the actin cytoskeleton, retrograde endocannabinoid signaling, and central carbon metabolism in cancer (Figure 1C). Gene functional annotation generated the following biological processes: cell cycle, aging, cell proliferation, cell migration, cell differentiation, apoptosis, axon guidance, hypoxia, actin cytoskeleton, and the MAP signaling pathway (Figure 1D). A Venn diagram was used to identify targets that overlapped among the downregulated miRNAs (miR139-3p, miR138-5p, miR24-2-5p, miR3593-3p, and miR29-2-3p). The results of the KEGG and gene ontology (GO) term analyses are provided in Supplementary Figure S1.

### 2.2. miRNA 363-5p and miRNA 335 Levels are Elevated in Rat Sciatic Nerves during the Postnatal Developmental Period and Decrease after Nerve Injury

We identified the most highly expressed microRNAs, such as miRNA 146, miRNA 363-5p, miRNA335, and miRNA29b-5p, and evaluated their expression during postnatal development and after sciatic nerve injury. miRNA 335 and miRNA 363-5p were highly expressed during postnatal development and were the most downregulated miRNAs after sciatic nerve injury (Supplementary Figure S2A,B). Therefore, we focused on miRNA335 and miRNA 363b-5p.

**Figure 2:**
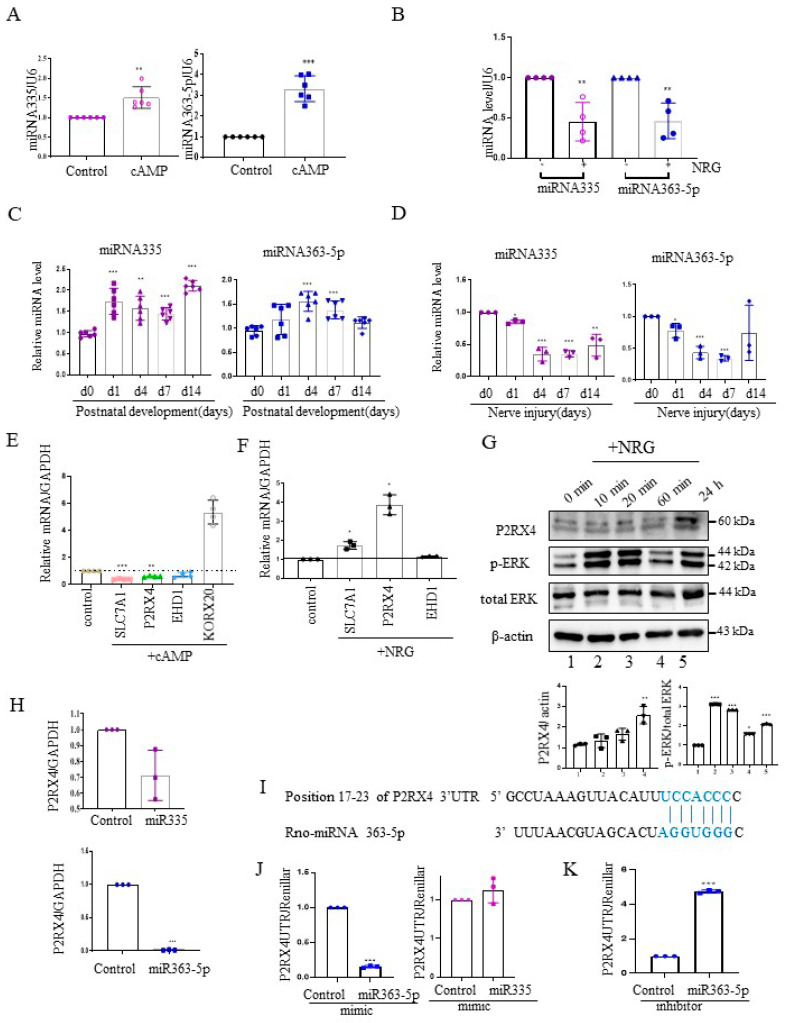
P2RX4 is a direct target of miRNA363-5p. (**A**) cAMP-induced Schwann cells exhibited an increased expression of miRNA335 and miRNA363-5p. *n* = 5. (**B**) NRG-treated Schwann cells exhibited a reduced expression of miRNA335 and miRNA363-5p. *n* = 4. (**C**) The expression levels of miRNA335 and miRNA363-5p increased during postnatal development. *n* = 6. (**D**) The expression levels of miRNA335 and miRNA363-5p decreased after sciatic nerve injury. *n* = 3. (**E**) The mRNA expression of predicted targets (slc7a1, P2RX4, and EHD1) in cAMP-induced Schwann cells. KROX2 was used as a positive control for cAMP. *n* = 4. (**F**) The mRNA expression of predicted targets (slc7a1, P2RX4, and EHD1) in NRG-induced Schwann cells. *n* = 3. (**G**) Upregulation of P2RX4 in NRG-induced Schwann cells. Schwann cells were treated with NRG, and total protein was collected for Western blot analysis. The lower panel shows the Western blot results. *n* = 3. (**H**) miRNA 363-5p inhibited the mRNA expression of P2RX4 in Schwann cells. Schwann cells were transfected with control, miRNA 363-5p, or miRNA 335 mimic (100 nM). At 48 h post-transfection, the mRNA level of P2RX4 was determined by RT-qPCR. *n* = 3. (**I**) Schematic representation of predicted functional interactions between miRNA 363-5p and seed sequences (bold) in the 3′-UTR of P2RX4 (http://www.targetscan.org/cgi-bin/vert_80/view_gene.cgi?rs=ENST00000337233.4&taxid=10116&members=&subset=1&showcnc=1&shownc=1&shownc_nc=1&showncf1=1&showncf2=1#miR-363-5p, accessed on 26 October 2021) (**J**,**K**) miRNA 363-5p regulates P2RX4 expression by directly targeting the P2RX4 3′-UTR in Schwann cells. Schwann cells were cotransfected with a pGL3-P2RX4-UTR reporter and miRNA 363-5p mimic (**J**, left), miRNA 335 mimic (**J**, right), or an miRNA363 inhibitor (**K**). *n* = 3. After 48 h, the luciferase activity of P2RX4 3-UTR was determined. Data are means ± SD. * *p* < 0.05, ** *p* < 0.01, *** *p* < 0.001.

Next, we investigated the expression of miRNA 335 and miRNA 363-5p from cAMP-induced Schwann cells using quantitative reverse transcription–polymerase chain reaction (RT-qPCR). miRNA335 and miRNA363-5p expression was increased in cAMP-induced Schwann cells (Figure 2A). Furthermore, their expression was elevated in rat sciatic nerves during the postnatal developmental period in vivo (Figure 2C). NRG contributes to the development of the peripheral nerve and the process of sciatic nerve injury [19]. NRG is an inducer of Schwann cell proliferation, and an important signal for maintenance of the myelin sheath during adulthood [20]. Therefore, we analyzed the expression levels of miRNA 335 and miRNA363-5p in NRG-induced Schwann cells. In contrast to cAMP-induced Schwann cells, miRNA335 and miRNA363-5p expression was downregulated in NRG-induced Schwann cells (Figure 2B). Sciatic nerve transection induced the expression of NRG in Schwann cells. Therefore, we measured the miRNA335 and miRNA363-5p expression after sciatic nerve injury. Similar to NRG stimulation in Schwann cells in vitro, the levels of miRNA 335 and miRNA363-5p were downregulated after sciatic nerve injury in vivo (Figure 2D).

To validate the miRNA target genes predicted based on Figure 1B, RT-qPCR was carried out with cAMP- and NRG-induced Schwann cells. The mRNA levels of solute carrier family 7 member 1 (Slc7a1), P2RX4, and EH domain-containing protein 1 (EHD1), which are predicted miRNA targets, were downregulated in cAMP-induced Schwann cells (Figure 2E), whereas P2RX4 and Slc7a1 levels were upregulated in NRG-induced Schwann cells (Figure 2F). KROX 20 was used as a positive control for cAMP stimulation. Consistent with this, Western blotting showed that P2RX4 was elevated in Schwann cells 24 h after NRG induction. P-ERK was used as a positive control for the NRG stimulation of Schwann cells (Figure 2G).

### 2.3. P2RX4 Is a Direct Target of miRNA 363-5p

Next, to determine whether P2RX4 was regulated by miRNA335 and miRNA 363-5p, we examined the level of P2RX4 after transfection with miRNA mimics in primary Schwann cells. Our results showed that miRNA 363-5p significantly suppressed the expression of P2RX4, but miRNA 335 was not significantly altered (Figure 2H). Therefore, we focused on miRNA 363-5p for further analysis. Using bioinformatics prediction software, we found that miRNA 363-5p could bind to the 3′-UTR of P2RX4 (Figure 2I). To determine whether miRNA 363-5p directly targets P2RX4, we performed a 3′-UTR reporter assay. As shown in Figure 2J, P2RX4-3′-UTR luciferase activity after miRNA 363-5p mimic transfection was downregulated through direct binding to the 3′-UTR (Figure 2J, left), but P2RX4-3′-UTR luciferase activity was not altered by miRNA 335 (Figure 2J, right). In addition, an miRNA363-5p inhibitor enhanced the luciferase activity of P2RX4 3′-UTR (Figure 2J,K). The results suggested that miRNA 363-5p regulates P2RX4 during Schwann cell proliferation and differentiation.

### 2.4. P2RX4 Is Elevated in Sciatic Nerve Injury

**Figure 3:**
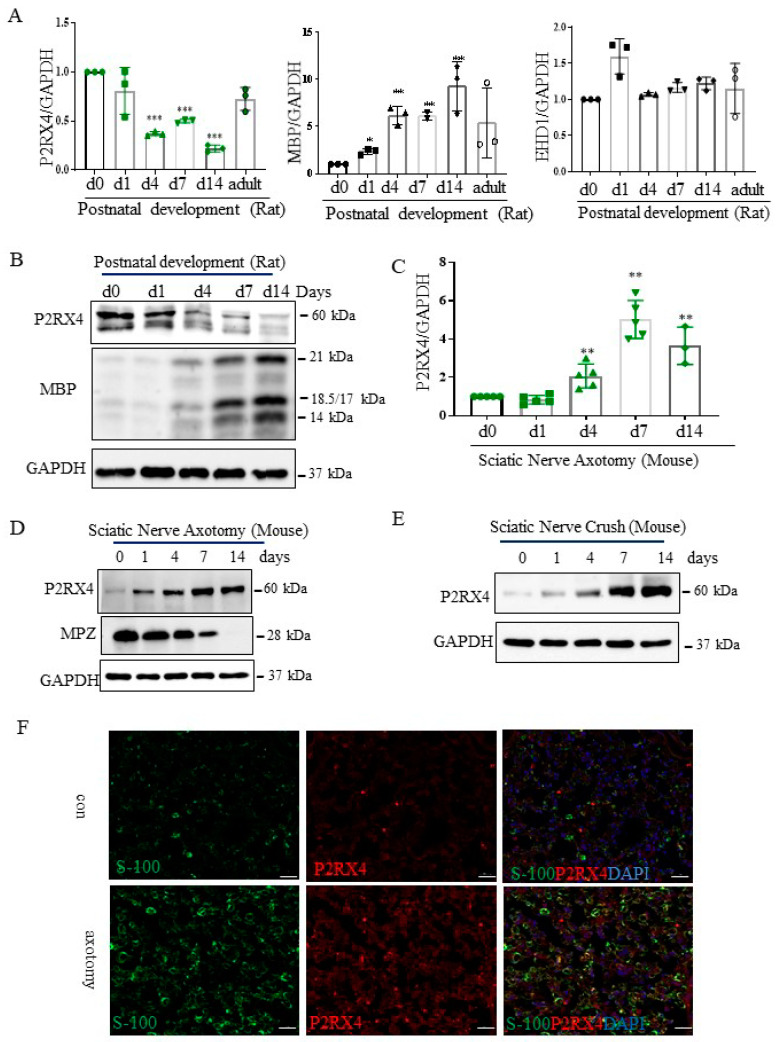
P2RX4 as differentially regulated during postnatal development and after sciatic nerve injury in vivo. P2RX4 mRNA ((**A**), *n* = 3) and protein (**B**) levels were downregulated during postnatal development. MBP was used as a positive control. P2RX4 mRNA ((**C**), *n* = 5) and protein (**D**) levels increased after axotomy. (**E**) The P2RX4 protein level decreased after mouse crush injury. Data are the mean ± SD. * *p* < 0.05, ** *p* < 0.01, *** *p* < 0.001. (**F**) Colocalization of P2RX4 (green) and S100 (red) 7 days after sciatic nerve injury. A transverse section of a mouse sciatic nerve was stained. Scale bar = 20 μm.

To assess the role of P2RX4 in the differentiation of the sciatic nerve in vivo, the expression of P2RX4 was measured during postnatal development from birth to the adult stage. As shown in Figure 3A, P2RX4 mRNA was downregulated in rat sciatic nerves during the postnatal developmental period. MBP was used as a positive control, and EHD1 was used as a negative control. Consistent with the detected mRNA levels, Western blotting showed that P2RX4 levels decreased from PO to P14 during rat postnatal development (Figure 3B). Although rats are typically used for sciatic injury models [21], mouse models has become increasingly common due to the varying availability of transgenic lines [22,23]. Therefore, we used a mouse model of sciatic nerve and crush injury.

**Figure 4:**
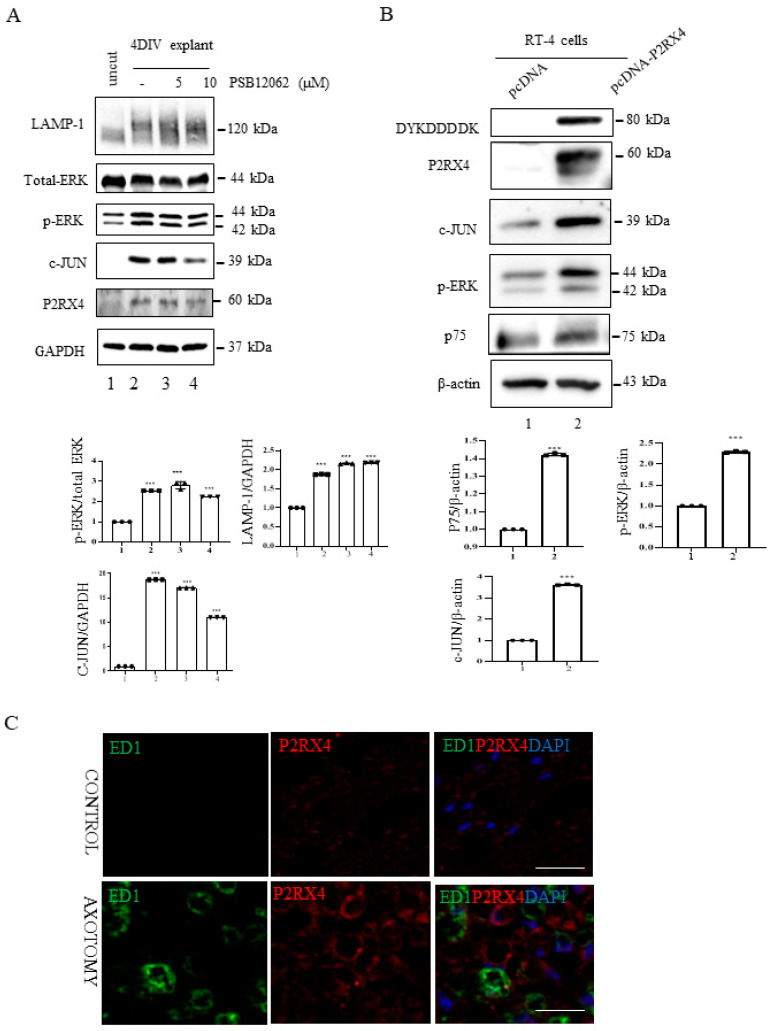
P2RX4 regulates p-ERK and c-JUN. (**A**) Sciatic nerve explants cultured for 4 DIV after P2RX4 antagonist (PSB1062) treatment exhibited decreased c-JUN and pERK levels. After the explants had been cultured, LAMP, ERK, c-JUN, P2RX4, and GAPDH proteins were collected for Western blotting. Uncut indicates freshly isolated sciatic nerves. The lower panel shows the Western blot results. *n* = 3. (**B**). Overexpression of P2RX4 by transfection with a pcDNA-P2RX4-DYKDDDDK-tagged plasmid enhanced the expression of pERK and c-JUN in RT4 Schwann cells. The lower panel shows the Western blot results. *n* = 3. Data are the mean ± SD. *** *p* < 0.001. (**C**) Immunofluorescence signals of P2RX4 (red) and ED1 (green, macrophage marker) in the sciatic nerve at 7 DIV. Scale bar = 20 μm.

Next, to investigate the role of P2RX4 in nerve regeneration, the expression of P2RX4 was examined after sciatic nerve injury. As shown in Figure 3C,D, RT-qPCR and Western blotting showed that P2RX4 was elevated at 4, 7, and 14 days after sciatic nerve axotomy. Consistently, P2RX4 was increased at 7 and 14 days after the nerve crush injury (Figure 3E). Interestingly, P2RX4 was present in two bands in rats and one in mice, as revealed by Western blotting. Immunofluorescence analysis was used to determine where P2RX4 localizes in Schwann cells. As shown in Supplementary Figure S3, endogenous S100 (Schwann cell marker; green) was colocalized with endogenous P2RX4 (red) after sciatic nerve injury. Macrophages are activated after nerve injury [24]. Therefore, to determine whether P2RX4 colocalizes in macrophages, immunofluorescence analysis was performed using sciatic nerves from 7 days in vitro (DIV). As shown in Figure 4C, P2RX4 did not colocalize with ED1, a macrophage marker (Figure 4C). This suggests that P2RX4 is associated with the dedifferentiation of Schwann cells during the period of Wallerian degeneration after sciatic nerve injury.

### 2.5. P2RX4 Regulates c-JUN and p-ERK Signals after Injury

In vitro explant cultures of mouse sciatic nerve were used to investigate the role of P2RX4 in Wallerian degeneration. Increased activation of ERK1/2 and c-JUN negatively regulates myelination [25,26]. Therefore, we investigated whether P2RX4 regulates ERK and c-JUN after injury. c-JUN and p-ERK in sciatic nerve explants cultured for 4 days were blocked by the inhibition of P2RX4 activity using a P2RX4 antagonist (PSB12062; 10 μM) (Figure 4A). N-(p-Methylphenylsulfonyl)phenoxazine (PSB12062) is a selective P2RX4 antagonist [27].

Furthermore, P2RX4 overexpression using pcDNA-P2RX4-DYKDDDDK-tag in RT4 Schwann cells enhanced p-ERK and c-JUN activity (Figure 4B). In addition, to determine whether P2RX4 is involved in Schwann cell migration, we performed a wound assay. Overexpression of P2RX4 promoted Schwann cell migration (Supplementary Figure S4). These results suggest that the upregulation of P2RX4 after sciatic nerve injury might be involved in the migration of Schwann cells.

### 2.6. P2RX4 and miRNA 363-5p May Be Important Regulators of Myelin Protein Breakdown

**Figure 5:**
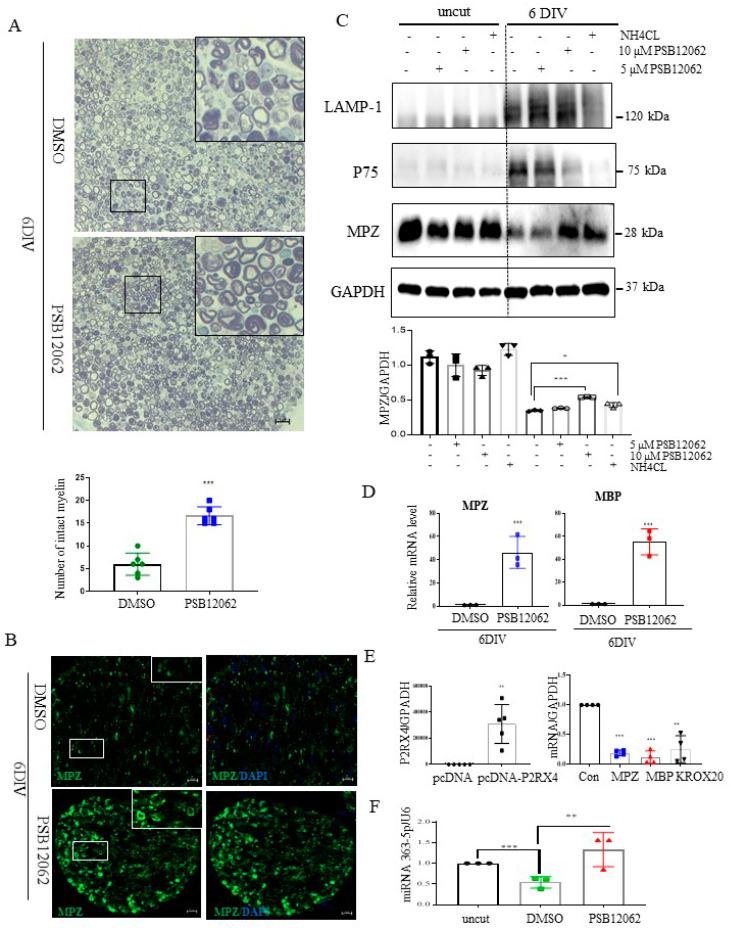
Sciatic nerve explants cultured for 6 DIV after P2RX4 antagonist treatment exhibited myelination upregulation. (**A**) Representative images of semithin sections showing that P2RX4 antagonist treatment (PSB12062) blocked the degradation of myelin proteins in nerve segments maintained for 6 DIV. Scale bar = 20 μm. The lower panel shows the number of intact myelin layers. *n* = 6. (**B**) Immunofluorescence analysis showing that the P2RX4 antagonist PSB12062 (10 μM) prevented the degradation of MPZ (green). Myelin was stained with MPZ (green, **left panel**) and nuclei with DAPI (blue, **right panel**). Scale bar = 50 μm. (**C**) Western blot showing that PSB12062 blocked degradation of the myelin protein MPZ in nerve segments maintained for 6 DIV. Uncut indicates freshly isolated nerves. Western blotting was carried out for LAMP1, p75, MPZ, and GAPDH. The lower panel shows the Western blot results. *n* = 3. (**D**) RT-qPCR analysis of *MPZ* and *MBP* mRNA levels in nerve segments maintained for 6 DIV treated with PSB12062, compared with the untreated control. Data are mean ± SD (error bars). *n* = 3. (**E**) Overexpression of P2RX4 by pcDNA-P2RX4-DYKDDDDK tag plasmid increased the MPZ, KROX20, and MBP levels. Left, level of P2RX4 after transfection with pcDNA or pcDNA-P2RX4-DYKDDDDK-tagged plasmid. *n* = 5. Right, levels of MPZ, KROX20, and MBP. *n* = 4. (**F**) RT-qPCR analysis of miRNA 363−5p expression in nerve segments maintained for 6 DIV and treated with PSB12062, compared with the untreated control. Uncut indicates freshly isolated nerves. *n* = 3. Data are the mean ± SD. * *p* < 0.05, ** *p* < 0.01, *** *p* < 0.001.

To assess whether P2RX4 is a regulator of degeneration after injury, a sciatic nerve segment was cultured for 6 days with the P2RX4 antagonist PSB12062. PSB12062 prevented the degradation of myelination in nerve segments maintained for 6 DIV (Figure 5A). Immunofluorescence analysis showed that MPZ expression was elevated in PSB12062-treated nerve segments at 6 DIV (Figure 5B). As shown in Figure 5C, Western blotting indicated that 10 μM PSB12062 prevented the degradation of MPZ expression, but it decreased the level of p75. LAMP1 was not altered. NH_4_Cl was used as a control to prevent the degeneration of myelin in explant cultures [28]. To determine whether the upregulation of myelin protein was due to transcriptional changes, we analyzed the mRNA levels of several myelin proteins, including MBP and MPZ, in nerve segments treated with the P2RX4 antagonist. Our results showed that the mRNA levels of the myelin proteins were enhanced in nerve segments treated with the P2RX4 antagonist compared with the control (Figure 5D). In contrast, overexpression from a P2RX4 plasmid in RT4 Schwann cells inhibited the mRNA levels of MBP and MPZ (Figure 5E). Interestingly, miRNA363-5p was significantly upregulated in nerve segments maintained for 6 DIV after P2RX4 antagonist (PSB12062) treatment compared with cut nerve segments, but it was downregulated compared with freshly isolated nerve segments (uncut) (Figure 5F). This indicates that P2RX4 and miRNA 363-5p are important regulators of the breakdown of myelin protein.

### 2.7. miRNA 363-5p and P2RX4 May Regulate Double-Negative Feedback during Wallerian Degeneration

**Figure 6:**
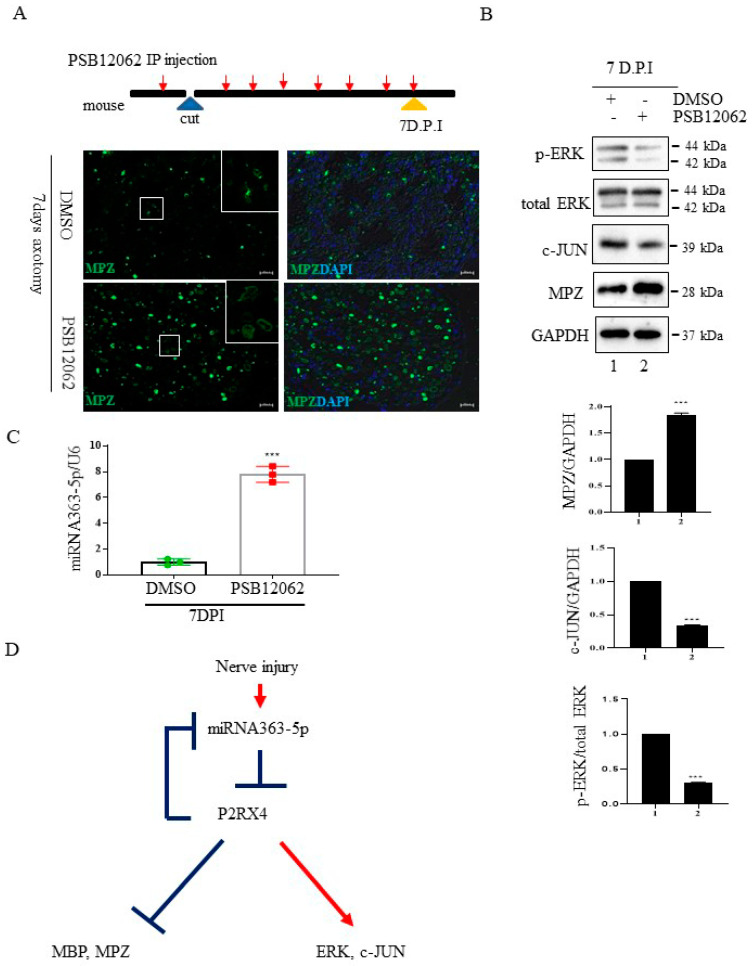
The P2RX4 antagonist PSB12062 blocked the degradation of myelination after nerve injury or during postnatal development in vivo. (**A**) Top, a schematic of P2RX4 antagonist administration after nerve axotomy. A P2RX4 antagonist (PSB12062) was intraperitoneally injected, once per day, from the day before nerve axotomy until 7 days postaxotomy. Bottom, immunofluorescence staining for MPZ (green) in sciatic nerves at 7 DPI compared with the control. Scale bar = 20 μm. (**B**) Western blot analysis of pERK and MPZ from the sciatic nerve of mice treated with PSB12062 and control (DMSO) after nerve injury. Lower panel shows the Western blot results. *n* = 3. (**C**) Levels of miRNA 363-5p from sciatic nerves 7 days after nerve injury and administration of the P2RX4 antagonist PSB12062 or control. *n* = 3. (**D**) Schematic of the double-negative feedback loop between miRNA 363-5p and P2RX4 after nerve injury. Data are the mean ± SD. *** *p* < 0.001.

To confirm that the inhibition of P2RX4 after nerve injury affects degeneration in vivo, we carried out sciatic nerve transection in 8-week-old adult mice and injected them intraperitoneally with a P2RX4 antagonist for 7 days. Immunofluorescence analysis showed that degradation of the myelin protein MPZ was blocked in the sciatic nerve at 7 days postinjury (DPI) after treatment with a P2RX4 antagonist (PSB12062) compared with the control (Figure 6A). Western blotting showed that the levels of p-ERK and c-JUN in sciatic nerve from mice injected with PSB12062 were reduced, whereas the level of MPZ was elevated compared with the control (Figure 6B). The level of miRNA363-5p was also upregulated in sciatic nerves from the PSB12062-treated group (Figure 6C). Our results suggest that miRNA363-5p and P2RX4 are involved in a double-negative feedback loop during Wallerian degeneration (Figure 6D).

## 3. Discussion

miRNAs may be potential biomarkers and therapeutic targets in various diseases. miRNAs contribute to peripheral nerve regeneration by affecting the proliferation and migration of Schwann cells [29], and they have been suggested as a potential therapeutic target for peripheral nerve repair [30,31]. In this study, we focused on the role of miRNA 363-5p in targeting P2RX4 during peripheral nerve regeneration via a double-negative feedback loop.

In this study, KEGG pathway enrichment and GO term analyses of the targets showed that the upregulated miRNAs were related to the calcium signaling pathway, regulation of the actin cytoskeleton, cell migration, response to ATP, and endocytosis, suggesting that they contribute to the phenotypic modulation of Schwann cells. Our data also showed that key factors such as P2RX5, guanine nucleotide-binding protein G(olf) subunit alpha (GNAL), P2RX4, P2RX3, platelet-derived growth factor receptor alpha (PDGFRA), and inositol 1,4,5-trisphosphate receptor type 2 (ITPR2; calcium signaling pathway) are potential targets of miRNAs regulating cAMP. Calcium signaling plays an important role in the development of the nervous system [32,33], and high concentrations of calcium inhibit Schwann cell frequency [34]. KEGG pathway enrichment and GO term analyses of the target downregulated miRNAs showed that they were related to the p53 signaling pathway, axon guidance, apoptosis, the Wnt signaling pathway, and the nuclear factor-kappa B signaling pathway. The Wnt/β-catenin signaling pathway play an important role in the development of neuropathic pain [35,36]. The transcription factor NF-κB regulates initial myelin formation during nerve development and remyelination following nerve injury [37,38].

The myelination status of Schwann cells is determined by the balance between opposing signals [25]. For example, Krox-20, OCT-6, and SOX10 maintain myelin differentiation, whereas Krox-20, OCT-6, and SOX10 negatively regulate dedifferentiation. c-Jun is upregulated after nerve injury, whereas c-Jun is normally suppressed as myelination [25]. Our data indicate that the upregulation of miRNA363-5p and miRNA335 during postnatal development enhances Schwann cell myelination, whereas the downregulation of miRNA 363-5p promotes the proliferation and migration of Schwann cells during nerve regeneration after injury.

The patterns of expression for central myelin genes differ markedly after nerve injury compared with during postnatal development. For example, MBP, Krox20, and MPZ are elevated during postnatal development, but suppressed after injury. In this study, the expression of miRNA-363-5p decreased and the expression of P2RX4 increased in sciatic nerve injury. In contrast, the expression of miRNA 363-5p increased and the expression of P2RX4 decreased during postnatal development. We also found that P2RX4 is a potential target of miRNA 363-5p.

Luciferase assays showed that miRNA 363-5p overexpression decreased P2RX4 levels, whereas the inhibition of miRNA 363-5p increased P2RX4 levels. Furthermore, overexpression of miRNA 363-5p inhibited the activity of P2RX4-UTR, whereas miRNA 363-5p inhibition led to increased P2RX4 UTR activity. Thus, our results suggest that P2RX4 is a direct target of miRNA 363-5p. Therefore, we focused on the possible regulatory role of miRNA 363-5p during peripheral nerve injury through the regulation of P2RX4.

Trophic factors and cytokines activate Schwann cells after damage. Degenerated Schwann cells in peripheral nerves express degeneration-associated genes, such as neurotrophic factors [39,40,41]. P2RX4 promotes TNF-α-induced brain-derived neurotrophic factor (BDNF) secretion in Schwann cells [42]. In this study, P2RX4 was elevated after sciatic nerve injury but downregulated during postnatal development. Treatment with PSB12062, a P2RX4 antagonist, blocked the degradation of myelin after sciatic nerve injury in explant culture in vitro. Intraperitoneal injection of PSB12062 after nerve injury also delayed myelin breakdown. These results indicate that P2RX4 may be an important regulator of dedifferentiation in Schwann cells.

Activation of ERK and c-JUN plays a key role in peripheral nerve injury. ERK phosphorylation is increased in sciatic nerve injury [43,44]. cAMP regulates neuregulin-dependent ERK/AKT signaling in SCs [45]. High levels of ERK1/2 activation negatively regulate myelination [25]. Furthermore, c-JUN negatively regulates myelination [26] and activates a repair program that triggers the regeneration of Schwann cells [46]. Although ERK is activated by growth factors or cytokines such as interleukin (IL)-1β and IL-6 after nerve injury, the mechanisms by which these factors stimulate the ERK pathway remain unknown. In this study, P2RX4 overexpression induced pERK in Schwann cells, and PSB12062 blocked pERK induction after 4 days of explant culture in vitro. Similarly, the intraperitoneal injection of PSB12062 attenuated ERK and c-JUN levels in sciatic nerves at 7 DPI. Collectively, the results of this study suggest that miRNA 363-5p regulates P2RX4 during nerve injury, and that P2RX4 contributes to the migration of Schwann cells by regulating ERK and c-JUN signaling.

## 4. Materials and Methods

### 4.1. Cell Culture

Primary Schwann cells were obtained from the sciatic nerves of 1-day-old rats, as described previously [47]. Primary Schwann cells were maintained in Dulbecco’s Modified Eagle Medium (DMEM; Gibco, Grand Island, NY, USA) containing 1% fetal calf serum (Invitrogen, Carlsbad, CA, USA), 10 μM forskolin (FSK; Sigma-Aldrich, St. Louis, MO, USA), 100 IU/mL penicillin, 100 g/mL streptomycin (Sigma-Aldrich), and 200 ng/mL neuregulin-1 (NRG-1; R&D Systems, Minneapolis, MN, USA) at 37 °C with 5% CO_2_. RT4 Schwann cells (RRID:CVCL_4006; Manassas, VA, USA) were cultured in DMEM containing 10% fetal calf serum (Invitrogen), 100 IU/mL penicillin, and 100 g/mL streptomycin (Sigma-Aldrich).

To assess the differentiation of cultured primary Schwann cells, 250 μM 8-(4-chlorophenylthio) adenosine-3′,5′-cyclic monophosphate (CPT-cAMP; Cat #A9501; Sigma-Aldrich) was added to primary Schwann cells and incubated for 72 h. For NRG-1 treatment, primary Schwann cells were serum-starved overnight and treated with NRG-1 (50 ng/mL; R&D Systems) for 1 day.

### 4.2. Sciatic Nerve Injury

Six-week-old C57BL/6 mice were purchased from Orient Bio Inc. (Seongnam, Korea) and maintained on a 12 h light/dark cycle. The room temperature was kept at 23 ± 1 °C with food and water provided ad libitum. Sex differences were not examined. Sciatic nerve axotomy and crush injury in adult mice (C57BL6) were conducted using protocols approved by the Dong-A University Committee on Animal Research. For nerve axotomy, sciatic nerves were sectioned using fine iris scissors (FST, Foster City, CA, USA). For crush injury, the sciatic nerve was crushed by squeezing firmly for 30 s with forceps (FST).

### 4.3. Peripheral Nerve Development

Pregnant Sprague Dawley rats (Orient Bio Inc.) were housed under a 12 h light/dark cycle and maintained at 23 ± 1 °C with food and water provided ad libitum. The day of birth was considered as postnatal day 0 (P0). Sex differences were not examined. Sciatic nerve segments were collected on P0, 1, 4, 7, and 14.

### 4.4. Explant Culture

Sciatic nerves from adult mice were cut into explants 3 mm in length. After removing the epineurium layer, the explants were maintained in DMEM containing 10% fetal bovine serum, 100 IU/mL penicillin, and 100 g/mL streptomycin (Sigma-Aldrich) at 37 °C with 5% CO_2_.

### 4.5. Semithin Section Analysis

Analysis of semithin sections of the sciatic nerves after injury was performed as described previously [48]. Briefly, after fixation, semithin (0.5 mm) sections were cut using an ultramicrotome and stained with toluidine blue. The sections were examined in bright-field mode using a Zeiss ApoTome 2 fluorescence microscope (Zeiss, Jena, Germany).

To count myelinated axons, images at high magnification were captured at regular intervals and centered within every third frame of the slot-mesh to replicate the conditions of an unbiased counting frame. In the calibrated images, axons were counted within the sampling frame and the density was estimated. The mean myelinated axon density was calculated from three individual images of each section. Myelinated axons were quantified by multiplying the mean axon density by the cross-sectional area of the specimen.

### 4.6. Intraperitoneal Administration of the P2RX4 Antagonist PSB12062

On the day of cutting the nerve, defined as day 0, 100 μM P2RX4 antagonist (PSB12062 (N-(p-methylphenyl)sulfonylphenoxazine); Sigma-Aldrich (Cat # 55476-47-6)) was administered before nerve injury. The same dose was administered daily from days 1 to 7 after injury (scheme in Figure 6A). On day 7 after injury, mice were sacrificed, and distal nerves were collected.

### 4.7. Immunofluorescence Staining

After fixation, the tissues were blocked with blocking buffer (phosphate-buffered saline (PBS) containing 0.2% Triton X-100 and 2% bovine serum albumin) for 1 h. Primary antibodies against P2RX4 (1:500; Cat # ab134559; Abcam, Cambridge, UK; RRID: AB_2891248), ED1 (1:1000; Cat# MCA341R; Bio-Rad, Hercules, CA, USA; RRID: AB_2291300,), S100 (1:1000; Cat# ab34686; Abcam; RRID: AB_77779), p75 (1:1000; Cat# sc-271708; Santa Cruz Biotechnology, Santa Cruz, CA, USA; RRID: AB_10714958), and myelin protein zero (1:1000; Cat# sc-18533; Santa Cruz Biotechnology; RRID: AB_2250708) were added for 16 h at 4 °C. The antibodies are listed in Supplementary Table S2. After washing three times, tissues were incubated with an Alexa 488-(1:1000) or Texas Red-(1:1500) conjugated secondary antibody (Molecular Probes, Eugene, OR, USA) for 1 h at room temperature. DAPI (1:250; Thermo Fisher Scientific, Waltham, MA, USA) was added to visualize nuclei. The coverslips were adhered to glass slides with mounting medium (Gel Mount; Sigma-Aldrich) and stained sections were imaged using a Zeiss ApoTome 2 fluorescence microscope.

### 4.8. Transfection

For plasmid transfections, primary and RT4 Schwann cells were transfected with pcDNA and pcDNA-P2RX4-DYKDDDDK-tag (Clone ID-ORa08201; GenScript Biotech, Piscataway, NJ, USA) using Lipofectamine 3000 (Life Technologies, Carlsbad, CA, USA) following the manufacturers’ protocols. For miRNA transfection, 100 nM miRNA mimics and inhibitors were transfected into primary SC cells using Lipofectamine 3000 (Life Technologies).

### 4.9. miRNA Array

Primary Schwann cells were treated with or without 250 μM 8-(4-chlorophenylthio) adenosine-3′,5′-cyclic monophosphate (CPT-cAMP) (Sigma). After 72 h, total RNA from primary Schwann cells was extracted with TRIzol reagent (Invitrogen) according to the manufacturer’s protocol. To examine changes in miRNA expression in cAMP-treated Schwann cells, an miRNA array was carried out according to standard protocols described elsewhere [49].

### 4.10. Biological Pathway Analyses

To analyze the biological pathways related to the miRNAs, the Database for Annotation, Visualization, and Integrated Discovery was used for GO term and KEGG analyses. *p <* 0.05 was considered to indicate functional enrichment.

### 4.11. Luciferase Assay

To detect P2RX4-untranslated region (UTR) activity by miRNA363-5p by 3′UTR-reporter assay, the 3′UTR from P2RX4 was fused to the end of a luciferase reporter gene. Luciferase reporter assays were used to assess the transcriptional activity of target genes. After seeding primary Schwann cells, a mixture of 100 ng of P2RX4-UTR (GeneCopoeia, Rockville, MD, USA) and 50 nmol miRNA mimic or inhibitor (Genolution, Seoul, Korea) was cotransfected using Lipofectamine 2000 transfection reagent (Invitrogen). Two days after transfection, a dual luciferase reporter assay (GeneCopoeia) was carried out to determine the firefly and Renilla luciferase activities (Promega, Madison, WI, USA). Luminescence was detected using a Promega GloMax 20/20 luminometer by the luciferin/luciferase chemiluminescent method.

### 4.12. Real-Time Quantitative PCR

Total RNA from Schwann cells was extracted using TRIzol (Life Technologies) and reverse-transcribed using a cDNA transcription kit (Promega) according to the manufacturer’s instructions. RT-qPCR was performed using SYBR Green Premix Ex Taq (Takara Bio Inc., Otsu, Japan) with an Applied Biosystems real-time PCR System. The primers used for RT-qPCR are listed in Supplementary Tables S3 and S4. The quantification cycles (Cq) were analyzed for each gene, and gene expression levels were calculated relative to the housekeeping gene glyceraldehyde-3-phosphate dehydrogenase (GAPDH) using the following equation [50]: Q = 2 − ΔCq. The data were analyzed using GraphPad Prism 7.0 (GraphPad Software Inc., San Diego, CA, USA).

An MiRNA-X^TM^ RNA First-Strand Synthesis Kit (Cat. #638313; Clontech Laboratories, Mountain View, CA, USA) was used to investigate miRNA levels. RT-qPCR was carried out with MiRNA-X miRNA RT-qPCR SYBR, according to the manufacturer’s instructions. Relative miRNA levels were calculated using the formula 2-ΔCq, where ΔCq = Cq(target miRNA) − Cq(U6). U6 (RNU6A) was used as the reference control.

### 4.13. Western Blotting

For tissue analysis, sciatic nerves were collected and mashed using lysing matrix beads (Metal Bead Lysing Matrix; MP Biomedicals, Santa Ana, CA, USA) in lysis buffer [51]. Schwann cells were harvested and lysed in RIPA buffer. After obtaining cell or tissue lysates, the proteins were separated by sodium dodecyl sulfate–polyacrylamide gel electrophoresis (SDS-PAGE). After transferring the proteins onto a nitrocellulose membrane (Amersham Biosciences, Little Chalfont, UK), the membrane was blocked with 0.1% Tween-20 and 5% nonfat dry milk in Tris-buffered saline (TBST) at room temperature for 1 h. Subsequently, the membrane was incubated with antibodies against P2RX4 (1:1000; ab134559; Abcam; RRID: AB_2891248), MBP (1:1000; AB980; MilliporeSigma, Burlington, MA, USA; RRID: AB_11211843), MPZ (1:500; sc-18533; Santa Cruz Biotechnology; RRID: AB_2250708), c-JUN (1:1000; sc-74543; Santa Cruz Biotechnology; RRID: AB_1121646), Lamp1 (1:500; sc-19992; Santa Cruz Biotechnology; RRID: AB_2134495), DYKDDDDK-tag (1:1000; A00187-200; GenScript; PRID: AB_1720813), GAPDH (1:1000; ab181603; Abcam; RRID: AB_2687666), and β-actin (1:1000; A5441; Sigma-Aldrich; RRID: AB_476744) in TBST at 4 °C overnight. After washing three times with 0.1% Tween-20 in PBS, the membranes were incubated with horseradish peroxidase (HRP)-conjugated secondary antibodies for 1 h, and the signal was examined by enhanced chemiluminescence (GE Healthcare Biosciences, Piscataway, NJ, USA). Actin may be associated with sciatic nerve injury [52,53]; therefore, we used GAPDH as a reference gene for all animal studies

### 4.14. Annotation of miRNA Targets

To identify the targets of the miRNAs, we used miRWalk database (http://mirwalk.umm.uni-heidelberg.de/, accessed on 26 October 2021)

### 4.15. Statistical Analysis

All the analyses were carried out at least three times; data are expressed as means ± standard deviation of the mean (SD). Statistical comparisons were performed using Student’s *t*-tests and one-way ANOVA (Figure 2C,D) in GraphPad Prism software.

## 5. Conclusions

cAMP-induced differentiated primary Schwann cells altered miRNA expression. *KEGG* pathway enrichment analysis of the target genes showed that upregulated miRNAs (miR212-5p, miR335, miR20b-5p, miR146b-3p, and miR363-5p) were involved in the calcium signaling pathway, the regulation of actin cytoskeleton, retrograde endocannabinoid signaling, and central carbon metabolism in cancer. Several key factors, such as P2RX5, GNAL, P2RX4, P2RX3, PDGFRA, and ITPR2 (calcium signaling pathway), represent potential targets of miRNAs regulating cAMP. miRNA363-5p was downregulated following sciatic nerve injury, whereas P2RX4 was upregulated. miRNA363-5p was upregulated during postnatal development, whereas P2RX4 was downregulated. miRNA363-5p may negatively regulate P2RX4 expression by promoting peripheral nerve regeneration, whereas miRNA 363-5p may positively regulate P2RX4 during postnatal development. In addition, miRNA363-5p overexpression negatively regulated P2RX4 by directly targeting its 3′-UTR. Administration of a P2RX4 antagonist in explant culture or the intraperitoneal injection of a P2RX4 antagonist after mouse sciatic injury blocked the degradation of MBP, and increased the expression of pERK and c-JUN. miRNA 363-5p regulated MBP, MPZ, and ERK by targeting P2RX4 during Wallerian degeneration, whereas the inhibition of P2RX4 increased the miRNA363-5 level, indicating that P2RX4 and miRNA363-5p reciprocally regulated each other in a double-negative feedback loop during Wallerian degeneration. The data provide insights into the negative feedback loop between miRNA 363-5p and P2RX4 in sciatic nerves.

## Data Availability

The datasets are available from the corresponding author upon reasonable request.

## Supplementary Materials

The following are available online at www.mdpi.com/1422-0067/222/11/1601/s1.
