# Supplementary material for: Involvement of the miR-363-5p/P2RX4 Axis in Regulating Schwann Cell Phenotype after Nerve Injury
Source: Int J Mol Sci. 2021 Oct 27;22(21):11601. doi: 10.3390/ijms222111601 (PMC8584002; doi:10.3390/ijms222111601)
Supplement: Supplementary file 1 [file ijms-22-11601-s001.zip › supplementary table2.3.pdf]

Supplementary table S2. Antibodies used in this study were listed

**Name**	**Immunogen**	**Source**	**Catalog number**	**RRID**
Anti-P2RX4	Synthetic peptide corresponding to Mouse P2X4 aa 56-69	Abcam	ab134559 Goat polyclonal	AB_2891248
Anti-MBP	Human myelin basic protein from brain	Sigma	AB980 Polyclonal antibody	AB_11211843
Anti-MPZ	peptide mapping near the C-terminus of zero of human origin.	Santac ruz	sc-18533 Goat polyclonal antibody	AB_2250708
Anti-c-JUN	amino acids 1-79 of c-Jun of human origin.	Santa cruz	sc-74543 Mouse monoclonal antibody	AB_1121646
Anti-ED-1	Rat spleen cells	Bio-Rad	MCA341R Mouse monoclonal antibody	AB_2291300
Anti-Lamp1	NIH/3T3 mouse embryo fibroblast tissue culture cell membranes.	Santa cruz	sc-19992 Rat monoclonal antibody	AB_2134495
Anti-DYKDDDDK-tag	A synthetic peptide (DYKDDDDK) coupled to KLH	GenScript	Cat# A00187 Mouse monoclonal antibody	AB_1720813
Anti-GAPDH	Recombinant fragment	Abcam	ab181603 Rabbit monolconal	AB_2687666
Anti-β-actin	Recombinant fragment	Sigma	A5441 Mouse monoclonal antibody	AB_476744

Anti-S100	Full length native protein (purified)	Abcam	ab34686 Rabbit polyclonal	AB_777793
Anti-p75	NGFR p75 from A875 melanoma cells of human origin	Santa cruz	Sc-58567 Mouse monoclonal	AB_10714958

Supplementary Table S3. Mouse Primer sequences used for the RT-qPCR analysis

Genes (Mouse)	Forward (5'-3')	Reverse (5'-3')
P2RX4	CCC TTT GCC TGC CCA GAT AT	CCG TAC GCC TTG GTG AGT GT
KROX 20	TTGACCAGATGAACGGAGTG	ACCAGGGTACTGTGGGTCAA
MBP	TACCCTGGCTAAAGCAGAGC	GAGGTGGTGTTCGAGGTGTC-
MPZ	GGT CCT GAG TTC AAA TCC C	GAA CCT TCA TCT GTT GTT GG
GAPDH	CAT GGC CTT CCG TGT TCC TA	CCT GCT TCA CCA CCT TCT TGA T

Supplementary Table 4. Rat primer sequences used for the RT-qPCR analysis

Genes (Rat)	Forward (5'-3')	Reverse (5'-3')
P2RX4	TGC GAC TGG AAG ATG TGT TC	CAA GAG GGT GAA GTT TTC TGC
EDH1	TGA TGT TGA GTG GGT AGT TGG	TGT TAG GCA GCT TGG ACT TC
Krox20	CAG TAC CCT GGT GCC AGC TG	TGT GGA TCT CTC TGG CAC GG
MBP	GGCACGCTTTCCAAAATCT	CGGGATTAAGAGAGGGTCTG-
SLC7A1	AGA AGC TAA GTA TGC AGT GGC	CCA CGA AGG CAT AAA AGC AG
GAPDH	GAC ATG CCG CCT GGA GAA AC	AGC CCA GGA TGC CCT TTA GT
