# Supplementary material for: Involvement of the miR-363-5p/P2RX4 Axis in Regulating Schwann Cell Phenotype after Nerve Injury
Source: Int J Mol Sci. 2021 Oct 27;22(21):11601. doi: 10.3390/ijms222111601 (PMC8584002; doi:10.3390/ijms222111601)
Supplement: Supplementary file 1 [file ijms-22-11601-s001.zip › SUPPLME MATERIAL.pdf]

SUPPLEMENTARY FIGUE LEGNEDS

Supplementary Fig S1. Biological function of upregulated miRNAs in cAMP-treated Schwann cells. (A) A Venn diagram was used to identify targets that overlapped among five different upregulated miRNAs (miR139-3p, miR 138-5p, miR24-2-5p, miR3593-3p, and miR129-2-3p). *KEGG* pathway assignments (B) and GO term analyses (C) for the target genes encoding the highly expressed miRNAs (miR139-3p, miR 138-5p, miR24-2-5p, miR3593-3p, and miR129-2-3p).

Supplementary Fig S2. The expression levels of miRNA 246, miRNA 335, miRNA 20b-5p and miRNA 363-5p during postnatal development. n=3. (D)The expression levels of miRNA 246, miRNA 335, miRNA 20b-5p and miRNA 363-5p after sciatic nerve injury. n=3 (E). Data represent mean ± SD. *$p < 0.05$, ** $p<0.01$, ***$p < 0.001$.

Supplementary Fig S3. Colocalization of endogenous P2RX4 (green) and S100 (red) 7 days after sciatic nerve injury. Transverse section of a mouse sciatic nerve was stained. scale bar = 20 µm

Supplementary Fig S4. P2RX4 overexpression in RT4 Schwann cells enhanced Schwann cell migration. Representative wound healing images in scratch of Schwann cells transfected with P2RX-4 overexpression plasmid. Cell migration was photographed 16 h after scratching in the control or P2RX4 overexpressed Schwann cells.
