# Supplementary material for: Involvement of the miR-363-5p/P2RX4 Axis in Regulating Schwann Cell Phenotype after Nerve Injury
Source: Int J Mol Sci. 2021 Oct 27;22(21):11601. doi: 10.3390/ijms222111601 (PMC8584002; doi:10.3390/ijms222111601)
Supplement: Supplementary file 1 [file ijms-22-11601-s001.zip › suppplementary20210621.pptx]

## Slide 1
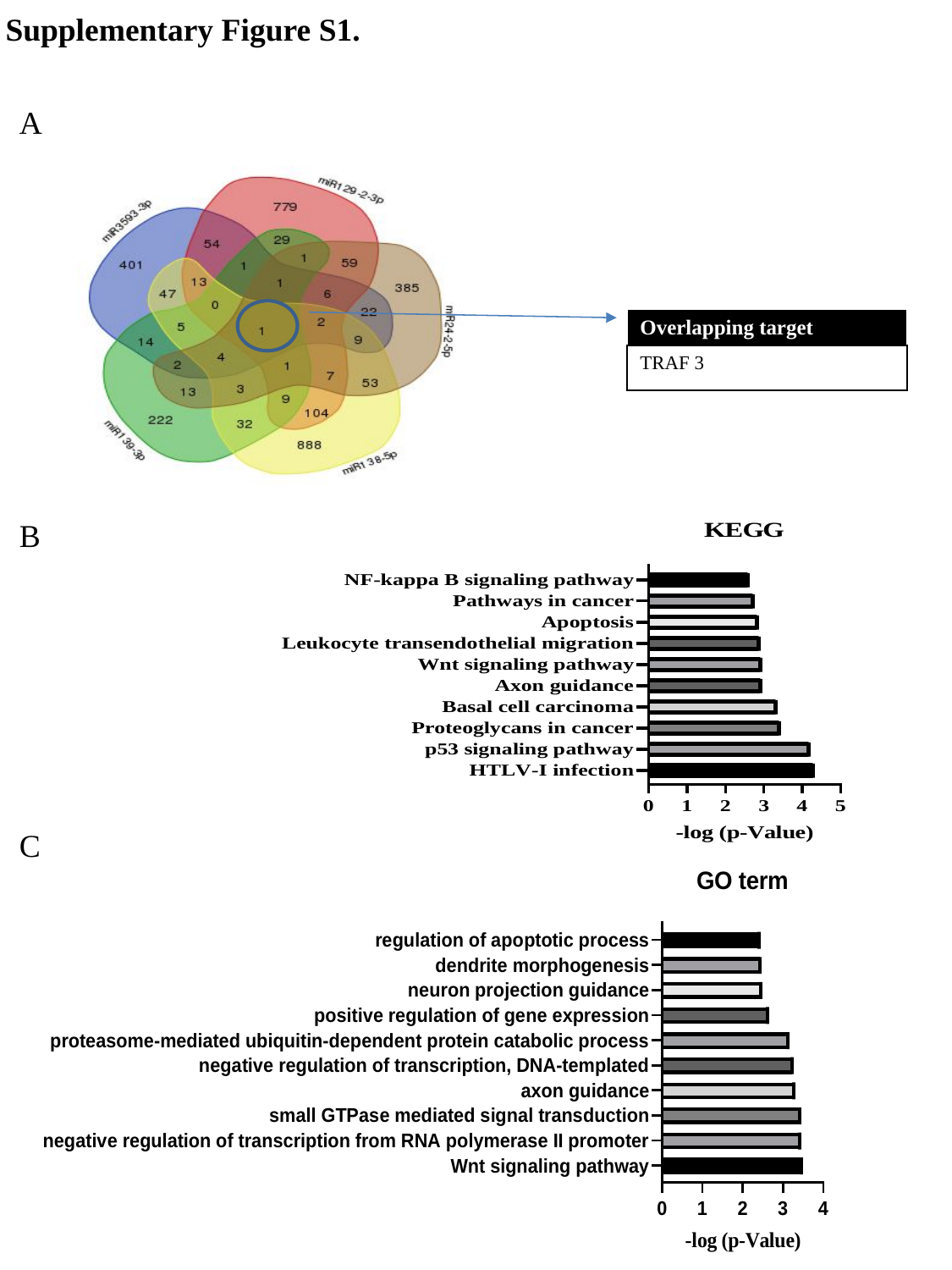

# Supplementary Figure S1.
A
| Overlapping target |
| --- |
| TRAF 3 |
B
C

## Slide 2
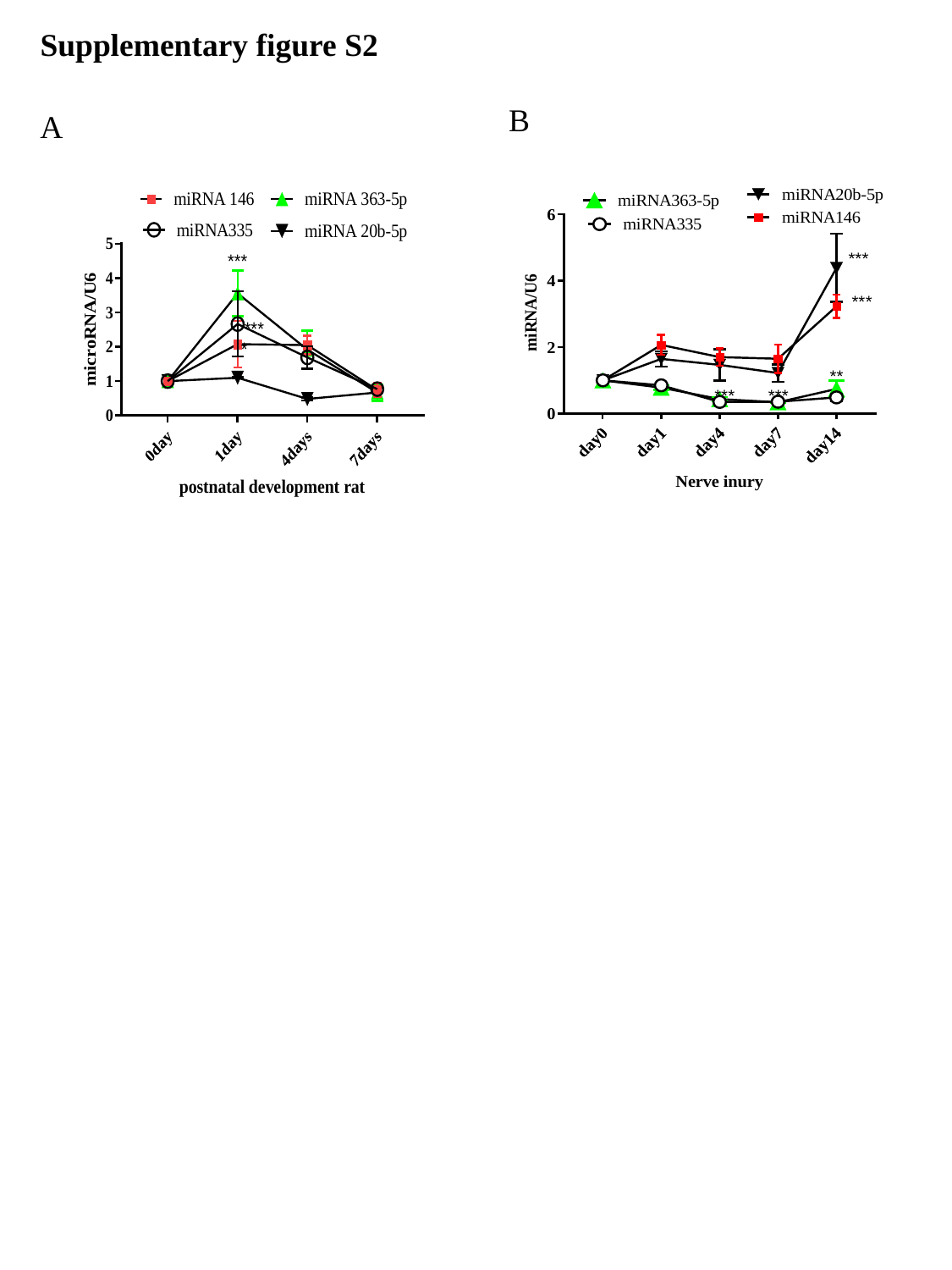

Supplementary figure S2
B
A

## Slide 3
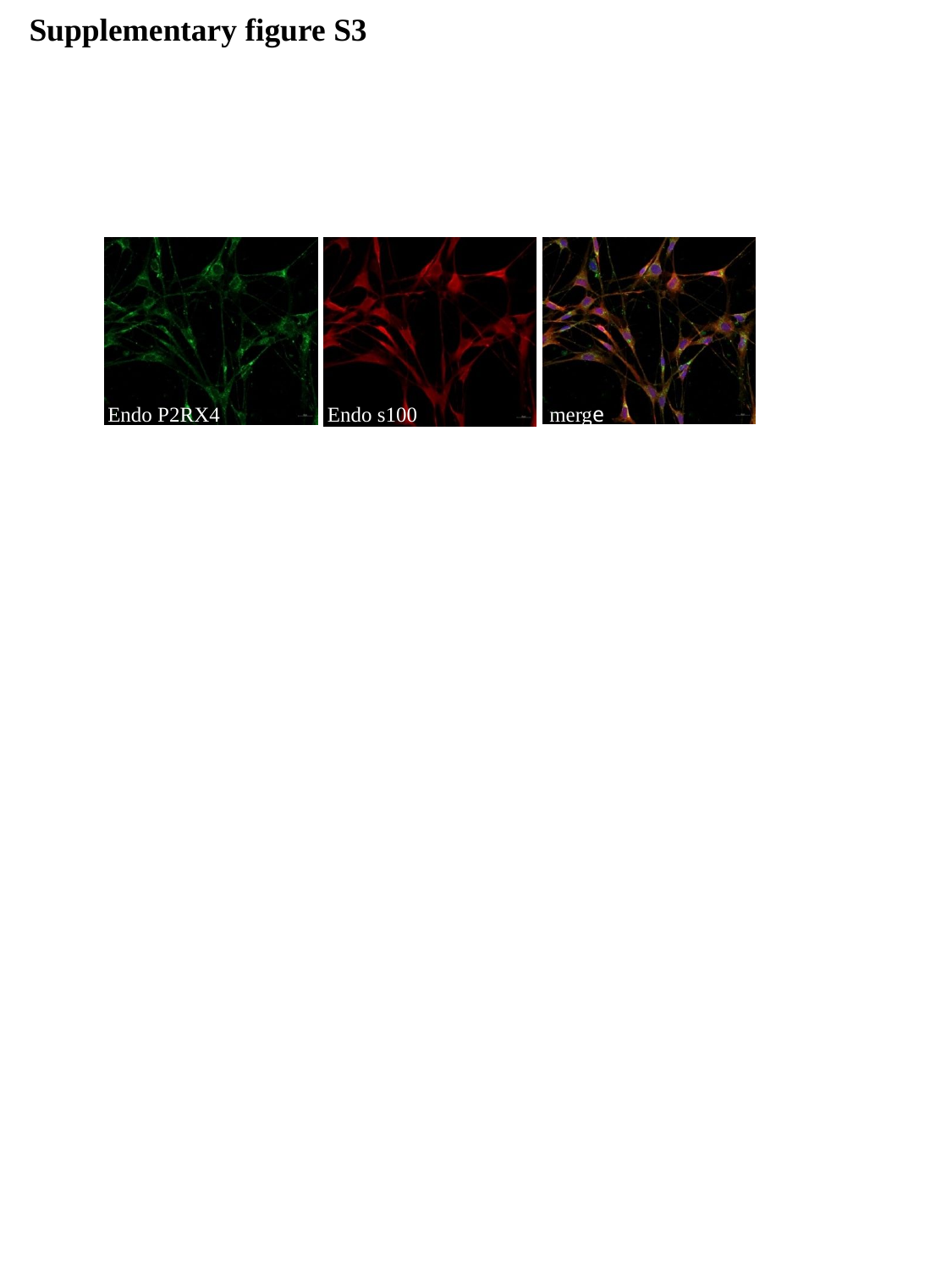

Supplementary figure S3
Endo s100
merge
Endo P2RX4

## Slide 4
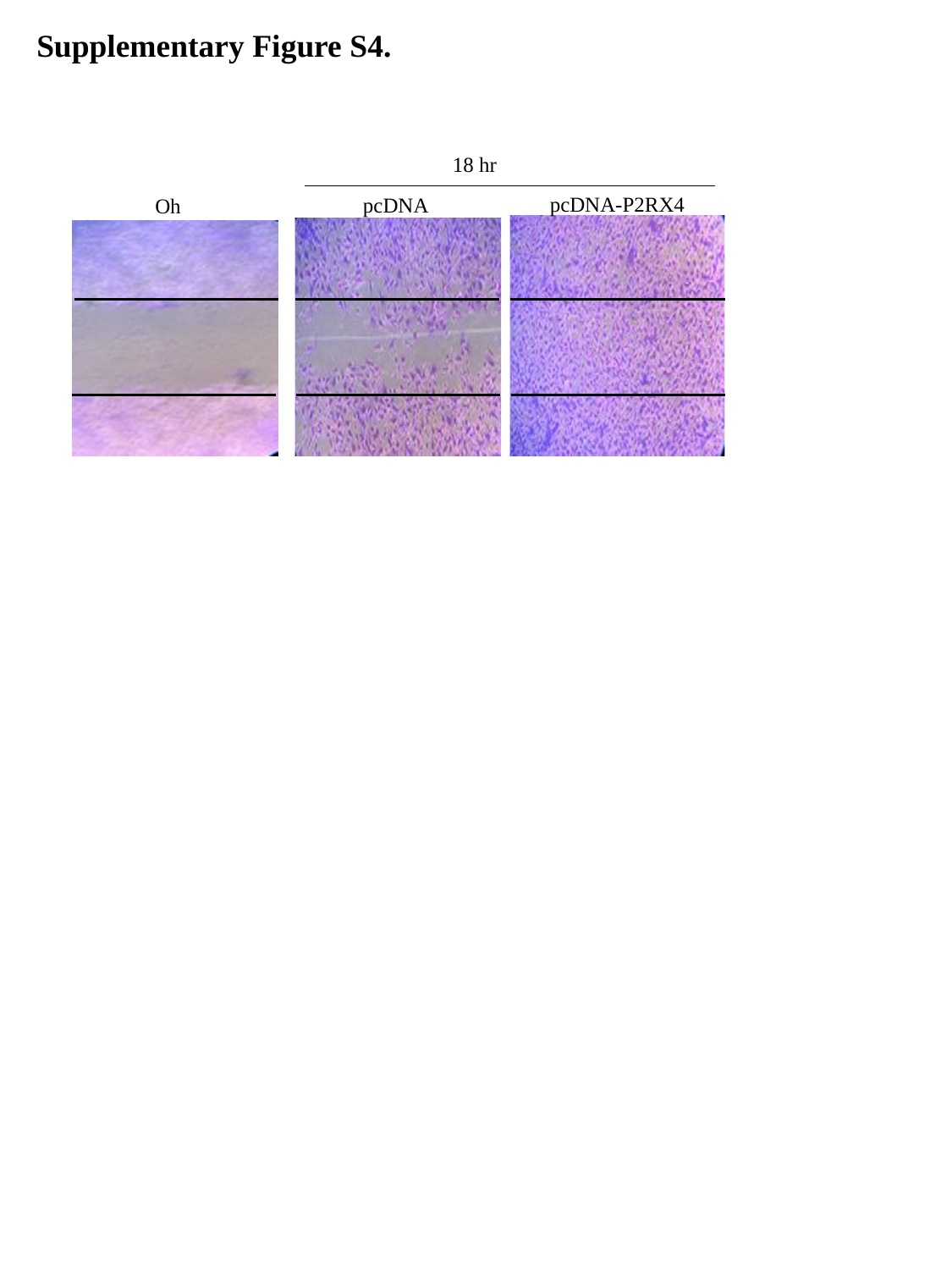

Supplementary Figure S4.
18 hr
pcDNA-P2RX4
pcDNA
Oh
